# Awareness of HPV and cervical cancer prevention among Cameroonian healthcare workers

**DOI:** 10.1186/1472-6874-11-45

**Published:** 2011-10-18

**Authors:** Catherine McCarey, David Pirek, Pierre Marie Tebeu, Michel Boulvain, Anderson Sama Doh, Patrick Petignat

**Affiliations:** 1 Geneva University School of Medicine, rue Michel-Servet, 1211 Genève, Switzerland; 2 Yaoundé University Faculty of Medicine, Mfoundi, Yaoundé, Cameroon; 3 Department of Obstetrics, Division of Development, Geneva University Hospitals, bld de la Cluse, 1211 Genève, Switzerland; 4 Yaoundé Gyneco-Obstetric and Pediatric Hospital, BP 4362, Yaoundé, Cameroon; 5 Department of Gynecology, Surgical Gynecologic Oncology Unit, Geneva University Hospitals, bld de la Cluse, 1211 Genève, Switzerland

## Abstract

**Background:**

Cervical cancer, although largely preventable, remains the most common cause of cancer mortality among women in low-resource countries.

The objective of this study was to assess knowledge and awareness of cervical cancer prevention among Cameroonian healthcare workers.

**Methods:**

A cross-sectional self-administered questionnaire in 5 parts with 46 items regarding cervical cancer etiology and prevention was addressed to healthcare workers in six hospitals of Yaoundé, Cameroon. The investigators enlisted heads of nursing and midwifery to distribute questionnaires to their staff, recruited doctors individually, in hospitals and during conferences and distributed questionnaires to students in Yaoundé University Hospital and Medical School. Eight hundred and fifty questionnaires were distributed, 401 collected. Data were analyzed with SPSS version 16.0. Chi-square tests were used and P-values < 0.05 were considered significant.

**Results:**

Mean age of respondents was 38 years (range 20-71 years). Most participants were aware that cervical cancer is a major public health concern (86%), were able to identify the most important etiological factors (58%) and believed that screening may prevent cervical cancer (90%) and may be performed by Pap test (84%). However, less than half considered VIA or HPV tests screening tests (38 and 47%, respectively). Knowledge about cancer etiology and screening was lowest among nurse/midwives.

**Conclusion:**

Knowledge of cervical cancer and prevention by screening showed several gaps and important misconceptions regarding screening methods.

Creating awareness among healthcare workers on risk factors and current methods for cervical cancer screening is a necessary step towards implementing effective prevention programs.

## Background

Worldwide, cervical cancer is the second most common cancer among women, after breast cancer. Every year, 500 000 new cases are diagnosed and 270 000 women die of this disease, mostly (85%) in developing countries [1]. The world pattern of cervical cancer indicates that this is predominantly a problem of low resource setting countries. The main reason is limited access to screening and treatment facilities [2]. Countries that have organized screening programs have substantially reduced cervical cancer incidence and mortality. Screening programs have the potential to be effective because cervical cancer is easily accessible to biopsy, there is a long latent period easily recognizable before development of cancer and there is an effective treatment in precursor disease.

In 2007, the Cameroonian population was estimated at 18 468 000 inhabitants, with an annual growth rate of 2.2% and crude birth rate of 35‰ [2]. About the half of the Cameroonian population is female (51%), and the life expectancy is about 53 years [3]. Women in their forties have on average 7 children and 21% of them have their first child when they are teenagers [3,4]. Approximately 6% of the population aged 15-49 is infected with HIV [5]. There is a national cervical cancer-screening program, but the service is limited to some main cities, and this probably contributes to the high incidence and mortality from cervical cancer in a country where 42% of the population is rural [2]. Based on hospital registry data, women most at risk for cervical cancer are those over 35 years with a median age at diagnosis of 49 years; most of them having an advanced and incurable disease at presentation [6]. The Cameroon Ministry of Health launched an awareness and educational campaign in the mass media and distributed information on the availability of cervical cancer screening. The method of screening was not specified but the tests (cytology-based or visual inspection with acetic acid (VIA)) were offered free of charge to the population.

Recent years have also seen significant developments in cervical cancer prevention. Previous efforts to implement cytology-based screening in developing countries have been conducted since the early 1980's. However, to date, they have failed to reduce the mortality rates mainly because of inadequate material resources, absence of a quality control system, lack of trained providers and of follow-up and treatment facilities.

Lack of knowledge about cervical cancer in the population and among healthcare workers is a prime barrier for access to cervical cancer prevention [6-8]. Our aim was to assess the knowledge and awareness of cervical cancer prevention among Cameroonian healthcare workers.

## Methods

The National Ethics Committee of Cameroon in Yaoundé approved the study and authorizations were obtained from hospital directors involved in the study. Between 1^st ^and 30 June 2009, 850 questionnaires were distributed and 401 collected in six public hospitals in Yaoundé (Yaoundé University Hospital, Central Hospital, General Hospital, Gyneco-Obstetric and Pediatric Hospital, Essos Hospital and Djoungolo Hospital). These were self-administered, anonymous, multiple-choice questionnaires, addressed to healthcare workers in contact with women (GPs, nurses, midwifes, pediatricians, gynecologists and obstetricians). Each participant received written explanations about the objectives of the study.

The investigators recruited most doctors and students individually, while nurses were contacted by head-nurses of the department. The questionnaire contained 46 multiple-choice questions (with « yes », « no », « I don't know » answers) covering the following topics: (i) knowledge of epidemiology of cervical cancer; (ii) risk factors; (iii) HPV infection and link to cervical cancer; (iv) screening methods and practices and (v) demographics and socio-professional questions. The data collected were analyzed using the statistical analysis program SPSS version 16.0. Chi-square tests were used and P-values < 0.05 were considered significant.

## Results

Median age of the respondents was 38 years; 95% of them were or had been sexually active. Other demographic characteristics of respondents are depicted in Table 1. Most respondents (86%) agreed that cervical cancer is one of the two most common cancers in Cameroonian women, and identified it as a major public health concern for the country.

**Table 1:** Demographic characteristics of respondents

Characteristics	N (%)
**Sex**	
Female	265 (66)
Male	136 (34)
**Age-group (years)**	
Median (range)	38 (20-71)
< 25	70 (17)
26 - 50	218 (54)
> 50	113 (28)
**Category of health workers**	
Medical students	71 (18)
Nursing-midwifery students	38 (10)
Doctors (GP, Ped, Ob-Gyn)*	58 (14)
Nurse-midwives **	234 (58)
**Marital status**	
Never married	175 (44)
Couple (includes free union, divorced, widowed)	226 (56)
**Children**	
None	136 (34)
< 4	142 (35)
≥ 4	123 (31)
**Condom use**	
Yes	170 (42)
No	166 (41)
Unknown	65 (17)
**Screening attitudes among women**	
Had not had a pap smear in the last 5 years	156 (59)

*GP: general practitioners; Ped: pediatricians; Ob-gyns: obstetricians/gynecologists (n = 13 (3% of all respondents)).

** Midwives: n = 20 (5% of all respondents)

The causative link between high-risk human papillomavirus (HPV) and cervical cancer was well identified by most respondents (Table 2). On the other hand, only 45% were aware that HIV infection is a risk factor for HPV infection and cervical cancer. Multiple sexual partners was correctly identified as being a risk factor by 71% of respondents, although fewer (44%) were aware of the logical follow-up to this, that a partner who has or has had many sexual partners is also a risk factor. Significant differences between doctors and nurse-midwives' knowledge on all of the items were observed (p < 0.05) (Table 3).

**Table 2:** Knowledge about HPV and vaccination

Variable	Total correct N (%)	Medical students N (%)	Nursing/Midwifery students N (%)	Doctors N (%)	Nurses/Midwives N (%)	P
HPV is identified in > 50% cervical cancers	242 (60)	57 (81)	16 (42)	47 (81)	122 (52)	0.000
HPV is a sexually transmitted disease	263 (66)	53 (76)	22 (58)	54 (93)	134 (57)	0.005
HPV vaccine helps prevent cervical cancer	177 (44)	42 (59)	12 (32)	41 (71)	82 (35)	0.000
Would recommend vaccine*	157 (89)	37 (88)	11 (92)	37 (90)	72 (88)	0.998

* Among those who believe that the vaccine can prevent cervical cancer.

**Table 3:** Awareness of risk factors, symptoms and screening methods of cervical cancer

Variable	Total correct N (%)	Medical students N (%)	Nursing/Midwifery students N (%)	Doctors N (%)	Nurses/Midwives N (%)	P
Knowledge of 4 most important risk factors^1^	231 (58)	48 (67)	16 (41)	52 (90)	115 (49)	0.000
Cervical cancer is preceded by treatable dysplasia	323 (81)	61 (86)	21 (54)	55 (95)	186 (79)	0.019
Dysplasia is usually asymptomatic	174 (43)	43 (61)	14 (38)	46 (79)	71 (30)	0.000
The following test may be used for screening:						
- Pap test^2^	337 (84)	58 (82)	29 (76)	54 (93)	196 (84)	0.777
- HPV test^3^	190 (47)	28 (39)	18 (47)	33 (57)	111 (47)	0.480
- VIA^4^	151 (38)	25 (35)	8 (21)	34 (57)	84 (36)	0.001

^1 ^HPV and HIV infections, multiple sexual partners and partner with multiple partners are the most important factors.

^2 ^Pap: Papanicolaou

^3 ^HPV: Human Papillomavirus

^4 ^VIA: visual inspection with acetic acid

Most respondents did not consider HPV a transient infection. Only 36% believed that HPV infection is most often cleared by a competent immune system and does not usually cause cancer. The more recent graduates were more likely to be aware of the immune system's ability to clear away infection than the older. Most respondents knew that cervical cancer is preceded by dysplasia, which can be treated to avoid progression towards cancer. However, approximately half considered that HPV infection and dysplasia are generally asymptomatic (see Table 3).

Pap smear was the most widely and consistently known screening method for cervical cancer. Other methods such as VIA or HPV testing were not recognized as such (Table 3). Screening uptake was surprisingly poor among healthcare workers more closely involved in women's health (59% of women had not had a pap smear in the last 5 years), (Table 1). Most healthcare workers (75%) believed the general Cameroonian population is not sufficiently informed about cervical cancer and screening. More than half (60%) of our respondents believed there is too little screening because campaigns are irregular (85% of gynecologists). Many respondents (54%) believed screening uptake in the general population is poor because it is too expensive (35% disagreed and 11% did not know).

Only 44% of respondents believed vaccination helps prevent cervical cancer. Most healthcare workers (75%) did not believe the vaccine had been proven to be effective yet, but 89% of those who did would recommend it for young women aged 10-25 years (Table 2).

## Discussion

This study was conducted at the same time as the Cameroon Ministry of Health's awareness and educational campaigns, with information on the availability of cervical cancer screening. Our purpose was to evaluate knowledge and awareness regarding cervical cancer screening among Cameroonian healthcare workers because identification of strengths and weaknesses in healthcare workers' knowledge may be crucial in targeted information campaigns.

We found the level of knowledge in our study sample to be comparable to studies of similar populations in other developing countries. A study conducted in Thailand in 2009 [9] found that 82% of nurses and 88% of doctors interviewed believed HPV to be a public health issue (as did 86% of our sample).

The Ministry of Health has informed the population that screening will be offered to women free of charge. However, cost is still an oft-cited reason for poor use of screening services by the general population. This is certainly a factor in rural settings, where cost of transport and time spent away from work add to the actual cost of screening. Offering more geographically accessible health facilities may effectively increase screening uptake [10]. Nevertheless, geographical access may only be a partial explanation, since among well-informed healthcare workers, for whom screening was free and provided in the workplace, uptake of these services was low, with more than 60% of women never having been screened (or not in the last 5 years). Other barriers to screening included "not feeling sick" [11,12] and "not feeling at risk" [12,13]. Although a positive association between women's knowledge about cervical cancer and the likelihood of her having a Pap smear has been found [14], poor uptake of screening services among the better-informed healthcare workers[12,13,15-18], and patients [19], was a recurrent finding in the studies reviewed and reasons for this should be further investigated. Recommendation of healthcare providers can also influence screening behaviour, and providers who lack basic knowledge may be a problem in this context [7].

Nurse-midwifes are generally the most visible, frontline personnel of the hospital and are crucial in providing health education to patients and general population. However, our data suggest that nurse-midwifes' levels of knowledge and understanding of cervical cancer as well as its preventable nature should be improved. For successful screening programs, all healthcare workers must understand the causative relationship between HPV and cervical cancer as well as the importance of screening as a preventive measure. Continuing nurse education may contribute to strengthening cervical cancer screening programs.

There is supporting evidence that direct visualization with acetic acid (VIA) may be a valuable alternative to Pap smears in areas where access to healthcare is a limitation [8,20]. However, more recent studies have shown that a single round of HPV testing might have a greater impact on the reduction of advanced cervical cancer cases and associated mortality than VIA or cytology [21]. With adequate infrastructure, HPV testing could be an attractive alternative to previously used screening methods, as it also requires fewer hospital visits, and could even be conducted by patients themselves.

HPV vaccine is a new concept among Cameroonian healthcare workers. Very few apart from the gynecologists interviewed were aware the vaccine exists. The inconsistencies in the respondents' opinions on HPV vaccination highlight the great confusion surrounding it, its use and limitations. When compared to the Thai cohort, the Cameroonian healthcare workers have a similar knowledge level on HPV, cervical cancer and awareness of its importance as a public health issue. The respondents in our study were slightly more likely to recommend HPV vaccination to their patients with 88% of nurses and 90% of doctors who would recommend vaccination. These figures were similar to those of a study of HPV vaccine acceptability conducted in Brazil [22], which found 82% of physicians and medical students would encourage HPV vaccination.

Notably this survey highlighted a lack of knowledge about the vaccine. Although most respondents reacted positively to the idea of vaccination against HPV, they expressed concern about the vaccine's efficacy. It would be important to ascertain how healthcare workers' opinion about the vaccine affects health, especially reproductive health.

## Conclusion

Among the stronger points of this study, it should be noted that this is the first study performed in Cameroon assessing knowledge of cervical cancer screening among healthcare workers, with a sample sufficiently large to draw significant conclusions and provide useful pointers for planning medical education for healthcare workers. The weaker aspects of the study are that the sample was not randomly selected, did not include rural health workers and the questionnaires were self-administrated; many of those distributed were not returned. One could therefore expect those who were less confident to have chosen not to answer. Overall, knowledge may be lower than appears in this research.

In conclusion, knowledge of cervical cancer and prevention by screening showed several gaps and important misconceptions. Continuing medical education programs including nurse-midwives should be conducted at the hospital level to spread knowledge about cervical cancer prevention. Healthcare workers including nurse-midwives should be trained to encourage screening. Finally, further research is needed to explain the reluctance of eligible healthcare workers to go for screening despite knowledge about the problem and ready access to screening facilities. Healthcare workers need to be targeted first because of their pivotal role in any future screening program.

## Competing interests

The authors declare that they have no competing interests.

## Authors' contributions

DP and CM designed the study protocol and questionnaires, collected data in Cameroon, created the data bank and carried out statistical analysis of data and wrote the article. PT and ASD supervised distribution of questionnaires and collection of data in Cameroon. MB provided substantial assistance with data bank creation and statistical analysis. PP designed and coordinated the study.

All authors read and approved the final manuscript.

## Pre-publication history

The pre-publication history for this paper can be accessed here:

http://www.biomedcentral.com/1472-6874/11/45/prepub
